# COX-2 overexpression increases malignant potential of human glioma cells through Id1

**DOI:** 10.18632/oncotarget.1370

**Published:** 2013-10-31

**Authors:** Kaiming Xu, Lanfang Wang, Hui-Kuo G. Shu

**Affiliations:** ^1^ Department of Radiation Oncology and the Winship Cancer Institute, Emory University, Atlanta, GA

**Keywords:** glioblastoma, glioma, brain tumor, tumorigenesis, cyclooxygenase-2, Id1

## Abstract

Increased COX-2 expression directly correlates with glioma grade and is associated with shorter survival in glioblastoma (GBM) patients. COX-2 is also regulated by epidermal growth factor receptor signaling which is important in the pathogenesis of GBMs. However, COX-2 expression has not been previously shown to directly alter malignancy of GBMs. Id1 is a member of the helix-loop-helix (HLH) family of transcriptional repressors that act as dominant-negative inhibitors of basic-HLH factors. This factor has been shown to be regulated by COX-2 in breast carcinoma cells and recent studies suggest that Id1 may also be involved in the genesis/progression of gliomas. We now show that COX-2 increases the aggressiveness of GBM cells. GBM cells with COX-2 overexpression show increased growth of colonies in soft agar. Tumorigenesis *in vivo* is also increased in both subcutaneous flank and orthotopic intracranial tumor models. COX-2 overexpression induces Id1 expression in two GBM cell lines suggesting a role for Id1 in glioma transformation/tumorigenesis. Furthermore, we find direct evidence of a role for Id1 with significant suppression of *in vitro* transformation and *in vivo* tumorigenesis in COX-2-overexpressing GBM cells where Id1 has been knocked down. In fact, Id1 is even more efficient at enhancing transformation/tumorigenesis of GBM cells than COX-2. Finally, GBM cells with COX-2 or Id1 overexpression show greater migration/invasive potential and tumors that arise from these cells also display increased microvessel density, results in line with the increased malignant potential seen in these cells. Thus, COX-2 enhances the malignancy of GBM cells through induction of Id1.

## INTRODUCTION

Cyclooxygenase-2 (COX-2) is an inducible isoform of cyclooxygenases that catalyzes conversion of arachidonic acid to prostaglandins and other eicosanoids [1]. COX-2 is overexpressed in many cancers and contributes to tumor development and progression [2-4]. Elevated COX-2 is associated with increased angiogenesis, tumor invasion and promotion of tumor cell resistance to apoptosis [5-8]. Previous studies revealed that COX-2 is overexpressed in many gliomas and expression level, in particular, is positively correlated with tumor grade [9, 10]. Furthermore, elevated COX-2 levels correlate with earlier recurrence and shorter survival in glioma patients [10].

Id proteins belong to the helix-loop-helix (HLH) family of transcriptional regulators, acting as dominant negative repressors of basic-HLH (bHLH) transcription factors [11-13]. Mechanistically, Id proteins dimerize with bHLH factors to repress their functions. Id proteins can inhibit various cellular processes including apoptosis and cellular differentiation. Four members of this family have been identified (Id1-4). These factors have both common and unique activities. With respect to its role in the development and maintenance of cancers, Id1 overexpression is seen in a variety of solid tumors and appears to affect a number of cellular processes associated with malignancies [14, 15]. In fact, high Id1 levels correlate strongly with poor prognosis, chemoresistance and tumor metastases. Id1 expression in human glioma specimens has also been reported and positively correlates with tumor grade [16]. Recently, glioma-initiating cells (GICs) have been found to display high levels of Id1 [17]. Moreover, TGF-β inhibitors were found in an animal model to prevent glioma initiation and recurrence by reducing Id1 expression and depleting this GIC population [17]. This work suggests that high Id1 levels may correlate with aggressive behavior and poor prognosis in glioblastoma patients. A more recent study found in a mouse model of gliomagenesis that a subpopulation of tumor cells displaying high levels of Id1 has high self-renewal capacity consistent with a role of Id1 in propagation of glioma stem cells [18].

While COX-2 expression is linked to more aggressive glioblastomas [10], this enzyme has never been directly demonstrated to affect the transforming/tumorigenic potential of glioma cells. In addition, COX-2 has been shown to regulate Id1 expression in a breast carcinoma line [19]. However, no further work has been reported this link in any other systems. In the present study, we report that COX-2 overexpression in human glioma cells increases their expression of Id1 protein and enhances their *in vitro* transforming and *in vivo* tumorigenic potentials. We further show that COX-2-dependent glioma transformation/tumorigenesis requires induction of Id1 and that Id1 overexpression also enhances glioma transformation/tumorigenesis. Finally, COX-2 and Id1 overexpression can both increase the invasive capacity of glioma cells and promote angiogenesis in xenograft tumors derived from these glioma cells.

## RESULTS

### Overexpression of COX2 in glioma cell lines leads to increased Id1 expression

**Figure 1:**
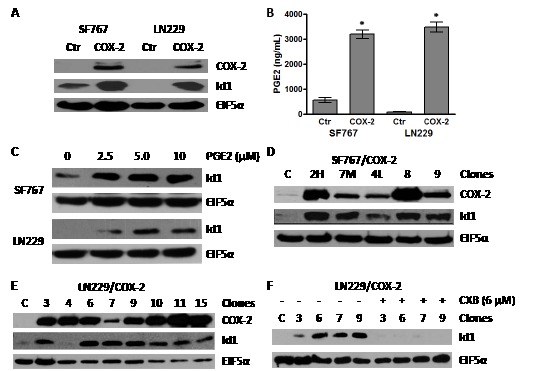
Overexpression of COX-2 leads to increase in Id1 protein level (A) Immunoblot analysis of the SF767 and LN229 glioma cells infected with retroviruses that express COX-2 cDNA. (B) PGE2 secreted by SF767/COX-2, LN229/COX-2 and respective control cells were determined by enzyme immunoassay. Production of PGE2 was determined in triplicate for each well with graph representing the average values (n=3)/cell line. *Error* bars are ± one standard error of the mean (SEM). * indicates statistically significant difference compared with control cells (*p* < 0.0001 in each case). (B) Immunoblot analysis of SF767 and LN229 glioma cells treated with PGE2. SF767 and LN229 cells were treated with the indicated amount of PGE2 for 24 hours. (D & E) Immunoblot analysis of different clones isolated from pools of SF767/COX-2 and LN229/COX-2, as indicated. (F) Immunoblot analysis of different LN229/COX-2 clones treated with the COX-2 inhibitor celecoxib (6 μM) for 24 hours. All blots were probed with antibodies against COX-2, Id1 and EIF5α (normalization control), as indicated, and represented results of 2-3 independent experiments.

A previous study revealed that COX-2-driven PGE2 induces expression of Id1 in breast cancer cells [19]. Therefore, we became interested in testing whether overexpression of COX-2 results in Id1 expression in glioma cells. To approach this question, we first infected SF767 and LN229 glioma cells with a retroviral expression vector containing COX-2 cDNA to generate pooled derivatives. These cells not only overexpressed COX-2 but also Id1 (Fig. 1A). To confirm that PGE2 production was increased in the COX-2 overexpressors, we measured PGE2 concentration by ELISA and found significantly increased levels in conditioned media of pooled COX-2-expressing SF767 and LN229 cells (Fig. 1B). Next, to establish whether PGE2 can induce Id1 in glioma cells, parental SF767 and LN229 cells were treated with increasing amounts of PGE2. As predicted, Id1 expression increased (Fig. 1C). We have now also established in multiple SF767 and LN229 COX-2 expressing clones, that in nearly every case, Id1 expression increases with COX-2 overexpression (Fig. 1D-E). To determine whether Id1 induction is truly dependent on COX-2 activity, four LN229/COX-2 clones were treated with the selective COX-2 inhibitor celecoxib (CXB) and assessed for Id1 expression by immunoblot analysis. In each case, elevated expression of Id1 was significantly reduced by CXB treatment (Fig. 1F).

### COX-2 and Id1 enhance transformation of glioma cells in vitro

**Figure 2:**
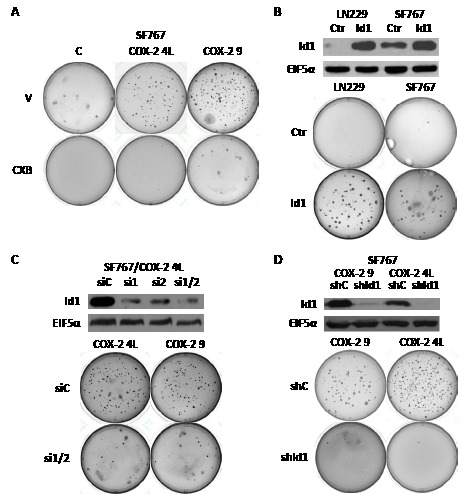
COX-2 overexpression induces soft agar colony formation through Id1. (A) SF767/control and two different SF767/COX-2 clones (4L and 9, see Fig. 1D) were assessed for growth in soft agar as described in Methods with vehicle (V) or celecoxib (CXB, 6 μM) and cultured for up to 30 days (until colonies were visible).(B) LN229 and SF767 were engineered to stably expressing human Id1 or control EGFP as confirmed by immunoblot analysis. Control and Id1-overexpressing cells were plated in soft agar and cultured until colonies were visible. (C) SF767/COX-2 clones (4L and 9) were transiently transfected with a control siRNA (siC) or two different siRNAs against human Id1 (singly or in combination; si1, si2 or si1/2) with Id1 knockdown in the 4L clone confirmed by immunoblot analysis. SF767/COX-2 clones (4L and 9) with (si1/2) or without (siC) Id1 knockdown were plated in soft agar and cultured until colonies were visible. (D) SF767/COX-2 clones (9 and 4L) were stably infected with a retroviral vector containing a control (shC) or Id1-targeting (shId1) shRNA with Id1 knockdown confirmed by immunoblot analysis. The SF767/COX-2 clones with (shId1) or without (shC) shRNA Id1 knockdown were plated in soft agar and cultured until colonies were visible. All blots were probed with antibodies against Id1 and EIF5α (normalization control), as indicated, and represented results from two independent experiments. For all soft agar experiments, representative images of plates from three independent experiments are shown.

To evaluate if COX-2 overexpression affects transformation of human glioma cells, we first performed soft agar colony formation assay using SF767/control cells and two SF767/COX-2 clones (4L and 9, see Fig. 1D) which express differing levels of both COX-2 and Id1. Both clones formed significantly more colonies in soft agar than corresponding control cells (Fig. 2A). We next sought to determine whether increased *in vitro* transforming potential was due to COX-2 activity. This soft agar assay was therefore repeated in the presence of the COX-2 inhibitor CXB and led to near abolishment of colony formation by SF767/COX-2 cells (Fig. 2A). Since we hypothesized that Id1 is a factor downstream of COX-2 enhancing its ability to alter transformation of glioma cells, we next assessed whether Id1 overexpression leads to increased soft agar colony formation. Expression vectors containing Id1 and corresponding control were introduced into the LN229 and SF767 glioma cells and immunoblot analysis confirms significant Id1 overexpression (Fig. 2B). Assessment of Id1 overexpressors and corresponding vector only controls revealed a significant increase in the colony forming capabilities of glioma cells expressing Id1 (Fig. 2C). To prove that Id1 is a critical downstream factor important in COX-2-mediated transformation, we next assessed growth in soft agar after knockdown of Id1 expression in SF767/COX-2 cells. Two siRNAs targeting different regions of the Id1 gene were transiently introduced into SF767/COX-2 cells either individually or together and showed significant suppression of Id1 expression (Fig. 2C). A representative clone 4L immunoblot is shown in Fig. 2C but similar results were obtained with clone 9 (data not shown). Since the greatest knockdown was noted with a combination of the two siRNAs, this mixture was introduced into SF767/COX-2 cells prior to seeding into soft agar. In each case, Id1 knockdown resulted in significant decreases in the formation of soft agar colonies (Fig. 2C). We next sought to achieve long-term suppression of Id1 in preparation for *in vivo* analysis by infecting our glioma cells with a retroviral vector expressing shRNA against Id1. Immunoblot analysis showed that Id1 protein level was significantly suppressed in SF767/COX-2 cells following introduction of shRNA vector (Fig. 2D). Consistent with the results described above, colony formation was significantly reduced for both SF767/COX-2 clones compared to controls (Fig. 2D). These results show that *in vitro* transformation of glioma cells is increased by expression of both COX-2 and Id1 and that Id1 appears to be a critical factor downstream of COX-2 important for this process.

### COX-2 and Id1 enhance glioma cell tumor growth in vivo

**Figure 3:**
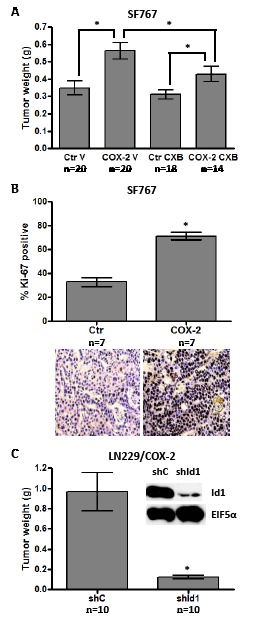
COX-2 overexpression promotes subcutaneous tumor growth through Id1 (A) SF767 control (Ctr) cells and COX-2 4L clones were subcutaneously injected (8x10^5^ cells/tumor) into mice (two tumors/mouse). Vehicle (V) or celecoxib (CXB, 150 mg/kg) was administered daily by oral gavage until tumor harvest. Tumors were dissected and weighed 28 days post- injection. Graph values are the average tumor weight ± one SEM. * denote statistically significant differences in mean tumor weight for the indicated comparisons by unpaired t-test (Ctr_V versus COX-2_V, p=0.001; Ctr_CXB versus COX-2_CXB, p=0.024; COX-2_V versus COX-2_CXB, p=0.050). (B) SF767/Ctr and SF767/COX-2 4L flank tumors were fixed and stained for Ki-67 to compare proliferation indices. Graph values are the average % Ki-67 staining ± one SEM (5 different high power fields with > 500 total nuclei were assessed for each tumor). * denotes a statistically significant increase in average % Ki-67 staining by unpaired t-test (Ctr versus COX-2, p < 0.0001). Representative fields for Ki-67 staining of the tumor types are shown below the graph. (C) LN229/COX-2 cells were infected with retroviruses encoding shRNA control (shC) or shRNA against human Id1 (shId1) and knockdown confirmed by immunoblot analysis. Blots were probed with antibodies against Id1 and EIF5α (normalization control), as indicated. The shC or shId1 cells were subcutaneously injected (2x10^6^ cells/tumor) into mice (two tumors/mouse). Upon sacrifice, tumor weights were determined and average values graphed ± one SEM. * denotes a statistically significant decrease in average tumor weight by unpaired t-test (shC versus shId1, p < 0.0005).

These encouraging results prompted us to further study the potential effect of COX-2 and Id1 overexpression on *in vivo* growth of glioma cells. We first chose to test LN229 glioma cells overexpressing COX-2 or Id1 along with the corresponding vector only control (Ctr) for growth as subcutaneous tumors on the flank of nude mice. Two different sets of experiments consistently showed that xenograft tumors derived from LN229/COX-2 and /Id1 cells grow significantly faster than their corresponding vector control (Table 1). In particular, Id1 overexpression had a stronger effect on the growth of LN229-derived tumors than COX-2. Next, we sought to confirm that COX-2 enhances growth of subcutaneous tumors in another glioma cell line (SF767). In this experiment, we also tested whether COX-2-mediated enhancement of tumorigenesis could be treated with the COX-2 inhibitor CXB. SF767/Ctr and /COX-2 (clone 4L) were injected into the flank of nude mice and allowed to grow for 28 days. Mice were treated with either vehicle only (V) or CXB (150 mg/kg/day by oral gavage) for duration of the experiment and tumors were dissected and weighed after 4 weeks of growth. As shown on the graph, COX-2 overexpression in SF767 significantly increased tumor growth in mice treated with vehicle only (Fig. 3A). Furthermore, CXB treatment reduced growth of COX-2 overexpressors but not corresponding control tumors (Fig. 3A). As expected, the proliferative indices of flank tumors derived from SF767/COX-2 cells were significantly higher than those of tumors derived from SF767/Ctr cells (Fig. 3B). Finally, we tested whether COX-2-mediated tumorigenesis also required Id1. The LN229/COX-2 line expressing either shId1 or corresponding control was again used. Expression of shId1 results in a significant knockdown of Id1 and, as is readily evident on the graph, a significant decrease in tumorigenic potential (Fig. 3C). Therefore, these results strongly suggest that COX-2 overexpression enhances not only *in vitro* transformation of glioma cells but also *in vivo* tumor growth in a process that is mainly mediated by Id1.

**Table 1:** 

Cell Type	Experiment #1*		Experiment #2†	
	Tumor weight, in gms (n)	Days post-injection	Tumor weight, in gms (n)	Days post-injection
LN229/Ctr	0.41 ± 0.07 (9)	93	0.45 ± 0.19 (9)	113
LN229/COX-2	1.99 ± 039 (14)	45	1.12 ± 0.39 (10)	55
LN229/Id1	3.25 ± 0.35(14)	35	1.67 ± 0.32 (8)	30

* Seeded with 4x10^6^ cells/tumor,

† Seeded with 2x10^6^ cells/tumor

**Figure 4:**
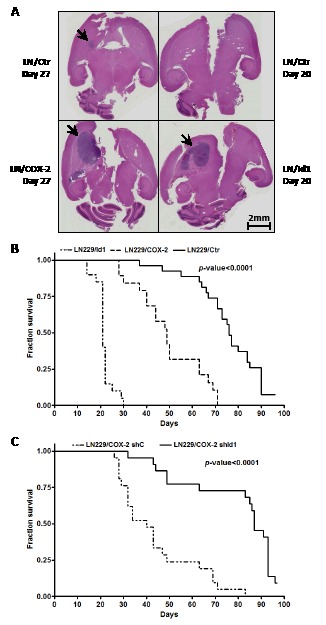
Intracranial tumor growth is enhanced by COX-2 and Id1 (A) Representative H&E stained coronal sections of mice brain harboring tumors derived from LN229/COX-2, LN229/Id1 and corresponding controls (harvested the indicated number of days post-injection) as described in the Methods section. Arrows denote brain tumors. No tumor was seen in our control mice harvested 20 days post-injection. Survival curves of mice injected intracranially with (B) LN229/Ctr (EGFP control, n = 27), LN229/COX-2 (n = 19) and LN229/Id1 (n = 20) or (C) LN229/COX-2 shC (n = 21) and shId1 (n = 22) (see Fig. 3B) are shown. Statistically significant differences between the survivals were found by log-rank test with p < 0.0001.

With increased tumorigenicity in a subcutaneous xenograft model, we next sought to verify that overexpression of both COX-2 and Id1 would also enhance tumorigenicity in an orthotopic tumor model. LN229 cells engineered to overexpress COX-2 and Id1 with corresponding control were intracranially injected (with 5x10^5^ cells) into nude mice and animals followed for survival. Brain tumors derived from LN229/COX-2 and LN229/Id1 cells were visibly larger than those derived from corresponding control cells harvested at the same time point (Fig. 4A). The median survival times of mice harboring LN229/Ctr cells (n = 27), LN229/COX-2 cells (n = 19) and LN229/Id1 cells (n = 20) were 76, 49 and 21 days, respectively, and the curves were significantly different (*p*-value < 0.0001) by log-rank test (Fig. 4B). To assess the role of Id1 in increased tumorigenic potential due to COX-2 in our orthotopic model, LN229/COX-2 expressing shId1 and corresponding control cells were injected intracranially and mice were again followed for survival. As expected, the control group (n = 22) had a median survival of 40 days which increased to 87 days with knockdown of Id1 (n = 21). This difference in survival was again significantly different (*p*-value < 0.0001) by log-rank test (Fig. 4C). Thus, both COX-2 and Id1 expression also enhances tumorigenicity of glioma cells in an intracranial tumor model.

### COX-2 and Id1 increase microvessel density in glioma xenograft tumors

**Figure 5:**
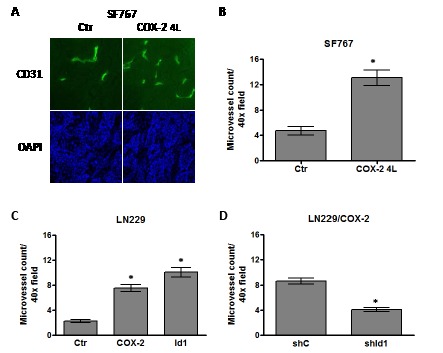
COX-2 overexpression increases microvessel density through Id1 Human glioma xenograft tumors were established in the flank of nude mice, harvested and immunofluorescently stained with antibody against CD31 to assess microvessel density. Microvessel density was quantitated by counting the number of CD31+ cells/40X objective field (minimum of 5 fields counted/tumor). (A) Representative fields are shown demonstrating CD31 staining (FITC, green fluorescence) in tumors derived from SF767/Ctr and SF767/COX-2 4L cells. Nuclei within each tumor were counterstained with DAPI (blue fluorescence). Bar graphs show the average microvessel count ± one SEM of xenograft tumors derived from (B) SF767/Ctr (n=10) and SF767/COX-2 4L (n=10) cells, (C) LN229/Ctr (n=9), LN229/COX-2 (n=8) and LN229/Id1 (n=8) cells or (D) LN229/COX-2 shC (n=10) and shId1 (n=9). * denote a statistically significant difference (at p < 0.05) when comparing with corresponding controls by unpaired t-test.

Our results above indicate that overexpression of both COX-2 and Id1 promotes the tumorigenic potential of glioma cells. Since microvascular proliferation is a hallmark of greater malignancy and a cardinal feature of glioblastomas, we were interested in determining whether COX-2 or Id1 overexpression will lead to increased angiogenesis. This was assessed by performing CD31 immunofluorescent staining of xenograft tumor samples and quantitating the microvessel counts. Microvessel density of flank tumors derived from SF767/Ctr and /COX-2 4L were initially compared. The COX-2-overexpressing tumors showed a higher level of staining with anti-CD31 antibody (Fig. 5A). Quantitation of microvessel density in the COX-2-overexpressing tumors revealed an average increase of 2.5-fold compared with the corresponding control tumors (Fig. 5B). We next assessed microvessel density in flank tumors derived from LN229 cells overexpressing COX-2 or Id1 along with corresponding control. Again, quantitation of vasculature in flank tumors derived from LN229/COX-2 and /Id1 cells revealed a significant increase in microvessel count in comparison with control tumors (Fig. 5C). Finally, similar to our results demonstrating a significant role for Id1 expression in COX-2-mediated transformation and tumorigenesis, we found that shRNA knockdown of Id1 in LN229/COX-2 cells results in about 60% decrease in microvessel density with this representing a statistically significant difference (Fig. 5D).

### COX-2 and Id1 promote migration and invasion of glioma cells

**Figure 6:**
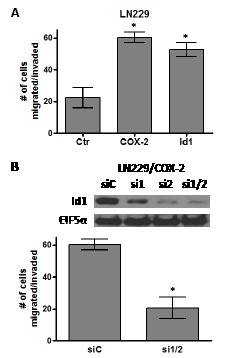
COX-2 overexpression promotes glioma cell migration through Id1 Bar graphs show the average number of cells that have migrated/invaded through the matrigel ± one SEM in our invasion assay (see Methods section). (A) LN229/Ctr, LN229/COX-2 and LN229/Id1 cells or (B) LN229/COX-2 transiently transfected with siRNA control (siC) and two siRNA (1 and 2) against Id1 (si1/2) are compared (n=3/cell type). * denote a statistically significant difference (at p < 0.05) when comparing with corresponding controls by unpaired t-test. Immunoblot analysis of LN229/COX-2 cells transfected with control siRNA (siC) and the two Id1 siRNAs (singly or in combination; si1, si2, or si1/2) demonstrate significant knockdown of Id1 after probing with antibodies against Id1 and EIF5α (normalization control).

Another feature important to the malignancy of glioblastomas is invasive capacity. COX-2 expression has been linked with an invasive cancer phenotype in multiple other systems. Therefore, we sought to determine whether COX-2 and Id1 overexpression will promote the migration and invasiveness of glioma cells. The migratory/invasive capacity of glioma cells was assessed by a Matrigel assay performed with Boyden chamber inserts in a 24-well plate as described in the Methods section. Forced expression of both COX-2 and Id1 in LN229 cells significantly enhanced invasive potential with migration through the Matrigel coated invasion chambers compared to the corresponding LN229 control cells (Fig. 6A). To assess the role of Id1 in COX-2-mediated migration/invasion, we suppressed Id1 expression in LN299/COX-2 cells with siRNAs against Id1 and then evaluated the ability of these cells to migrate through Matrigel invasion chambers. We again used the same two siRNAs to target Id1 that were used previously (Fig. 2C). These siRNAs could effectively knockdown Id1 expression in LN229/COX-2 cells when introduced both individually or together with most efficient suppression using the combination (Fig. 6B, upper panel). Repeat of the invasion assay with LN229/COX-2 cells transfected with either control (siC) or the two Id1 (si1/2) siRNAs and showed that knockdown of Id1 significantly reduced the ability of these cells to traverse through Matrigel in the invasion chamber (Fig. 6B, lower panel).

### Id1 overexpression enhances the formation of neurospheres from glioma tumors

**Figure 7:**
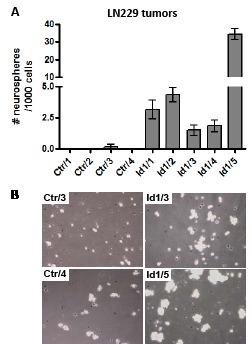
Id1 enhances formation of neurospheres derived from glioma tumors (A) Bar graphs show the average number of neurospheres ± one SEM that developed in each well after culturing 10^3^ cells/well in a 96-well dish for 7 days. Each value is the average of 6 wells from independent subcutaneous flank tumors that are derived from control (Ctr) or Id1-overexpressing LN229 cells. (B) Representative phase contrast microscopic images of cells from the indicated Ctr or Id1 tumors cultured under neural stem cell conditions after 3 days are shown. 10^5^ cells/well in a 24-well were seeded for these pictures.

Given the recent reports that Id1 plays a significant role in maintaining glioma stem cells [17, 18, 20, 21], we were interested in determining whether it would also do so in our tumor system. To assess whether Id1 enhances growth of glioma stem cells, we harvested flank tumors derived from LN229/Ctr and LN229/Id1 cells and made single cell suspensions that were then cultured in serum-free conditions designed to promote proliferation of neural stem cells within neurospheres. Formation of neurospheres from cells derived from these tumors was quantitated by determining the average number of neurospheres that grow out after 7 days when seeded with 1000 cells/well (on a 96-well plate). Under these conditions, almost no neurospheres developed from cells derived from LN229/Ctr tumors while neurosphere developed in nearly every well from cells derived from LN229/Id1 tumors (Fig. 7A). Representative pictures show clear formation of cellular clusters representing early neurosphere formation in tumors overexpressing Id1 while corresponding cultures of cells from control tumors do not show this clustering (Fig. 7B).

## DISCUSSION

In our current study, we find that overexpression of COX-2 in human glioma cells increases the malignant potential of these cells by enhancing expression of Id1. This conclusion is supported by several lines of evidence. First, forced expression of COX-2 in two human glioma cell lines leads to elevated expression of Id1 and the COX-2 inhibitor CXB dramatically inhibits the expression of Id1 in COX-2-overexpressing LN229 cells. Secondly, forced expression of COX-2 increases the malignancy of human glioma cells as evidenced by increased growth *in vitro* as colonies in soft agar and *in vivo* as xenograft tumors in nude mice and these effects are mainly mediated by Id1. Thirdly, COX-2 overexpression promotes important GBM phenotypes including tumor angiogenesis and invasive potential at least in part through induction of Id1. Finally, Id1 also enhances our ability to grow out GICs, or glioma stem cells as neurospheres in tumors that overexpress this transcriptional regulator.

The role of COX-2 in tumor angiogenesis is well-established [8, 22, 23] and specific inhibitors of this enzyme have been explored as part of anti-angiogenic “cocktails” used in therapy for various malignancies including glioblastoma [24-26]. In our current report, we clearly show that COX-2 expression is pro-angiogenic in gliomas with increased microvessel density in tumors derived from glioma cells engineered to overexpress COX-2 (Fig. 5). While the role of the Ids in angiogenesis is less recognized, this class of protein also has essential pro-angiogenic activities [27, 28]. In agreement with this, Id1 overexpression can also increase microvessel density in xenograft tumors derived from glioma cells overexpressing Id1 (Fig. 5B). More importantly, knockdown of Id1 expression in glioma cells overexpressing COX-2 reduced the microvessel count in tumors derived from such cells by roughly half (Fig. 5C). While defining the precise mechanism by which Id1 influences angiogenesis in glioblastoma is beyond the scope of this current study, some studies suggest potential factors involved in this process. Tanaka et al. found that E2-2, a basic HLH transcription factor, can suppress expression of vascular endothelial growth factor receptor 2 (VEGFR2) and Id1-mediated inhibition of E2-2 may enhance VEGFR2 expression and angiogenesis [29]. Additionally, Id1 can downregulate the angiogenic inhibitor thrombospondin-1 (TSP1) and promote neovascularization [30, 31]. Finally, Id1 has been linked with glioma stem cell propagation [17, 20, 21] which may contribute to increased angiogenesis since these stem-like cancer cells can differentiate in vascular endothelial cells promoting tumor angiogenesis [32-35].

The ability to infiltrate along white matter tracts is a hallmark feature of glioblastomas and remains a key reason why surgery is unable to completely eradicate this disease [36]. We found that overexpression of both COX-2 and Id1 in the LN229 glioma cell line results in a greater potential for migration in a matrigel invasion assay (Fig. 6A). This suggests that these factors may play an important contributory role in the glioblastoma invasion phenotype. Glioblastoma invasive ability has also been associated with certain angiogenic regulators [37, 38] linking these different phenotypes that are positively altered by COX-2 and Id1. Like its role in COX-2-mediated angiogenesis, Id1 is also critical for COX-2-mediated migration/invasion of glioma cells with a significant reduction in the migration of LN229/COX-2 cells when Id1 expression has been knocked down (Fig. 6B). Consistent with our findings, Id proteins has been shown to enhance tumor migration and invasion by upregulation of metalloproteinases and semaphorin 3F [39, 40].

As previously stated, COX-2 overexpression is linked with higher grade gliomas [9]. In addition, elevated levels of COX-2 have been shown to be an independent negative prognostic factor in glioblastoma patients [10]. We have also previously shown COX-2 regulation by signaling pathways arising from epidermal growth factor receptor accepted to be important in glioma pathogenesis [41-43]. These results suggest that COX-2 is important in the pathogenesis of glioblastomas and may be a good therapeutic target. Our current work further supports this idea with the direct demonstration that COX-2 enhances the malignancy of human glioma cells. The New Approaches to Brain Tumor Therapy (NABTT) consortium (now part of the Adult Brain Tumor Consortium) has tested the utility of CXB with standard radiation and temozolomide in the upfront treatment of glioblastoma patients and found overall survival to be on the order of what is seen in glioblastoma patients treated with just RT and temozolomide [44]. However, this was a relatively small study with no selection for glioblastomas with high COX-2 expression, the subgroup that would be most likely to respond to CXB therapy. Given our results, it may be reasonable to consider another trial exploring the utility of CXB in glioblastomas that display higher COX-2 expression.

Our identification of Id1 as a critical factor downstream of COX-2 important for its activity in glioma cells is particularly interesting and consistent with the work of others linking COX-2 with Id1. Lei et al. found potential association between Id1 and COX-2 for angiogenesis in gastric cancer [45]. Subbramiah et al. also found that COX-2-derived prostaglandin E2 can transcriptionally upregulate Id1 expression in breast carcinoma cells [19]. More recently, several groups have demonstrated links between glioblastoma malignancy and Id1. Anido et al. found that TGF-β signaling is linked to high Id1 expression in their glioblastoma models and specific inhibition of TGF-β in their models can reduce the glioma initiating cell (GIC) population through suppression of Id1 expression [17]. Niola et al. found that the Id family proteins can affect the aggressiveness of gliomas in a mouse tumor model and are needed to maintain GICs in their perivascular niche where they must reside to maintain self-renewal capabilities [20]. Finally, Soroceanu et al. found that Id1 increases the invasive phenotype in gliomas and can similarly enhance the aggressiveness of orthotopic tumors [21]. Our results are consistent with these previous reports and taken together suggest that the Id proteins may be a good therapeutic target in glioblastomas although development of effective inhibitors will be needed before this can be clinically tested.

In summary, we show that COX-2-dependent transformation of glioma cells is dependent on Id1 expression. While high COX-2 expression is clearly linked to increasing glioma grade and is associated with worse outcomes in patients with this disease [9, 10], to our knowledge, our report is the first to demonstrate that COX-2 has direct transforming activity by increasing the malignancy of glioma cells both *in vitro* and *in vivo*. We have also identified a novel link between COX-2 and Id1 in gliomas and demonstrated that Id1 is a critical factor downstream of COX-2 required for enhancing the aggressiveness of glioma cells. Finally, our work continues to add to the growing evidence that Id proteins are important in the aggressiveness of gliomas and demonstrates additional situations where Id protein expression can be regulated.

## MATERIALS AND METHODS

### Plasmids

The enhanced green fluorescent protein (EGFP) and rat COX-2 cDNAs were inserted downstream of the CMV promoter in an MuLV-based retroviral vector containing the neomycin resistance gene (pKX95) to create pKX139 and pKX104C, respectively, as described previously [46]. Human Id1 cDNA (OriGene Technologies Inc., Rockville, MD) was inserted downstream of the CMV promoter in the pKX95 to create pKX95/Id1. Retroviral vectors encoding human Id1 and control shRNAs (pSM2c-based system) were purchased (Open Biosystems, a subsidiary of Thermo Fisher Scientific Inc., Waltham, MA).

### Cell lines, culture conditions and transfections

Glioma cell lines (SF767, LN229, and their derivatives) were cultured in high glucose DME media (Sigma-Aldrich, St Louis, MO) supplemented with 10% fetal calf serum (Sigma), Na pyruvate (1 mM) and penicillin (100 U/ml)/streptomycin (100 μg/ml) unless otherwise indicated. Phoenix cells were cultured in the above media without Na pyruvate. Retroviral vectors were transiently transfected into Phoenix cell by CaPO_4_ precipitation. To generate glioma cell lines (clones and pools) stably expressing COX-2 or Id1, SF767 and LN229 cells were successively infected with conditioned media from transfected Phoenix cells and selected with 1000 μg/ml G418 (Invitrogen, Carlsbad, CA). For transient Id1 knockdown by siRNA suppression, SF767 and LN229 cells were transfected with siRNA against Id1 (Integrated DNA Technologies, Coralville, IA) using Lipofectamine 2000 (Invitrogen). The Stealth RNAi medium GC duplex-negative control (Invitrogen) was used to control for sequence-independent effects of introducing short RNA duplexes into cells. For stable Id1 knockdown, SF767/COX-2 and LN229/COX-2 cells were successively infected with conditioned media from the Phoenix cells transfected with the shId1 retroviral construct or corresponding control and selected with 2 μg/ml puromycin. For culturing under neural stem cell conditions, a single cell suspension was made by mincing the indicated subcutaneous tumors, treating with Collagenase/Dispase (1 mg/ml in PBS) (Roche Applied Science, Indianapolis, IN), passing through a 70 micron mesh and further isolating single cells on a Ficoll gradient. The indicated numbers of viable single cells were seeded in NeuroCult NS-A Basal Media with 10% NeuroCult NS-A Proliferation Supplement, Heparin solution (2 μg/ml), rh bFGF (10 ng/ml) and rh EGF (20 ng/ml) (STEMCELL Technologies, Vancouver, BC, Canada) for the indicated times. Standard aseptic culture techniques were used to propagate the various cells used in this report. All cell lines are periodically checked for mycoplasma infection by PCR (last tested in 2012). Cell line authentication by STR analysis was performed on 2/2013 for all parental glioma cell lines studied.

### Immunoblotting, immunohistochemistry, enzyme immunoassay and antibodies

All Western blots were done by standard procedures as previously described [41]. Blots were probed with antibodies against COX-2 (Cayman Chemical, Ann Arbor, MI) and Id1 (Santa Cruz Biotechnology, Santa Cruz, CA). As loading controls, blots were also probed with antibodies against EIF5α (Santa Cruz). Blots were detected using anti-rabbit or mouse IgG secondary antibody conjugated with horseradish peroxidase and chemiluminescent substrate according to standard procedures. Formalin-fixed, paraffin-embedded tumors were immunohistochemically stained for Ki-67 using a commercially available kit (Biocare Medical, Concord, CA) according to manufacturer's recommendations. Prostaglandin E2 (PGE2) levels were assessed by enzyme immunoassay. Cells of interest are seeded in 12-well plates, grown to 90% confluence and then cultured in fresh 300 μl serum-free medium for 24 hours. PGE2 released into this conditioned media was measured in triplicate by enzyme immunoassay following manufacture's procedure (Cayman Chemical) and normalized to protein content in lysates.

### Soft-agar colony-forming assay

Soft agar assay was performed in six-well plates by standard procedures. Briefly, a bottom layer consisting of 0.5% bacto agar in 1 ml of DME media supplemented with 10% FBS was first allowed to solidify in each well. The cells of interest (6 × 10^3^cells/well) were then mixed in 1 ml of the same media containing 0.3% bacto agar and this was allowed to solidify on top of the bottom agar media layer. An additional 1 ml layer of media with 0.3% bacto agar was allowed to solidify above the agar media layer that contained the cells. Each well was further supplemented with 0.5 ml of fresh DME media once a week until colonies have grown to appropriate size. The colonies were visualized after staining with 0.005% crystal violet.

### Tumor xenograft models

Animal experiments were approved by the Institutional Animal Care and Use Committee at Emory University. Flank and intracranial xenograft models utilized 4-5 week old female athymic nude mice (Harlan Laboratories, Indianapolis, IN). Flank tumors (2 sites/mouse) were seeded by subcutaneous injection of the indicated number of glioma cells in 200 μl of HBSS containing 10% matrigel (BD Biosciences, San Jose, CA). For intracranial inoculation, anesthetized nude mice were placed in a stereotaxic instrument and cells (5 × 10^5^ in 5 μl of HBSS) were stereotactically inoculated into the right striatum, 3 mm below the dural surface. Mice harboring tumor were housed and maintained to survival endpoints under specific pathogen-free conditions. Mice brains harboring tumors were harvested, formalin fixed/paraffin embedded, sectioned and stained with hematoxylin/eosin. Images were acquired using the Nanozoomer 2.0HT digital slide scanner (Hamamatsu Corporation, Bridgewater, NJ) and visualized on the Image Scope software (Aperio Technologies, Vista, CA). To evaluate the effect of COX-2 inhibition on COX-2 overexpressing flank tumors, celecoxib (150 mg/kg) or vehicle only (0.5% methylcellulose:10% PEG 200) was administered daily by oral gavage before tumor harvest.

### Microvessel density

Flank tumor frozen sections (5 μm) were fixed in cold methanol and stained with rat anti-mouse CD31 monoclonal antibody (BD Biosciences) as previously described [47]. CD31-stained slides were observed by fluorescent microscopy and images captured by an associated digital camera. Images were acquired and stored using the AxioVision software (Carl Zeiss Microscopy, Jena, Germany).

### Cell Invasion Assay

Invasion assay was performed using the 24-well Matrigel invasion chambers (BD Biosciences). 2.5 × 10^4^cells in 500 μl serum-free DME medium containing 1% BSA were loaded into the top inserts. DME medium with 1% FBS was added to the lower chamber (750 μl) as a chemoattractant. After incubation for 48 hours at 37°C, non-invasive cells were removed from the top of the Matrigel with a cotton-tipped swab. Invasive cells that migrated through 8 μm pores to the underside of the membrane were fixed and stained using Diff-Quick stain kit (IMEB Inc, San Marcos, CA) before counting under a microscope.
